# Conventional Versus New Treatment: Comparing the Effects of Acetylcholinesterase Inhibitors and N-Methyl-D-Aspartate Receptor Antagonist With Aducanumab

**DOI:** 10.7759/cureus.31065

**Published:** 2022-11-03

**Authors:** Elysia Chin, Ecler Jaqua, Mary Safaeipour, Tyler Ladue

**Affiliations:** 1 Family Medicine, University of California Los Angeles, Los Angeles, USA; 2 Family Medicine, Loma Linda University Medical Center, Loma Linda, USA; 3 School of Medicine, Loma Linda University School of Medicine, Loma Linda, USA

**Keywords:** cognitive impairment, cholinesterase inhibitors, nmda receptor antagonist, aducanumab, side effects of medical treatment, alzheimer’s dementia

## Abstract

Alzheimer’s dementia (AD) is the most common major neurocognitive impairment and the fifth leading cause of death in older adults in the United States. The diagnosis is clinical; however, laboratory tests and imaging frequently rule out secondary causes of dementia. Unfortunately, the treatment available for AD does not reverse dementia, but it may help improve the symptoms and slow the progression of the disease. The conventional treatment - acetylcholinesterase inhibitor (AChEI) therapy and N-methyl-D-aspartate (NMDA) receptor antagonist - is considered to enhance executive function, overall cognition, and activities of daily living. AChEIs such as donepezil, rivastigmine, and galantamine are approved for mild-to-moderate dementia. Furthermore, memantine, an NMDA receptor antagonist, is authorized for moderate-to-severe dementia. Aducanumab, the newest drug available, is an amyloid-beta (Aβ) monoclonal antibody approved only for mild AD. Treatment with either AChEIs or memantine is more cost-effective than aducanumab and the best supportive care. Aducanumab has particular recommendations with strict monitoring and several adverse effects, including amyloid-related imaging abnormalities. The most common adverse effects of AChEIs and memantine include gastrointestinal symptoms, dizziness, confusion, and headaches. Therefore, monitoring should be performed periodically at the clinician’s discretion for clinical response and tolerability of medication. Conventional therapies are only for symptom management but are still beneficial to patients and caregivers. Unfortunately, at this time, aducanumab’s risks outweigh the benefits with a questionable approval process by the Food and Drug Administration (FDA). However, given the potential disease-modifying capabilities of aducanumab, other disease-modifying options may become available by possibly reducing inflammation, preventing Aβ plaques from clumping, or keeping tau proteins from tangling.

## Introduction and background

Major neurocognitive impairment, also known as dementia, is a neurodegenerative disorder not explained by delirium or psychiatric disorder and is characterized by the abnormal deposition of amyloid and tau proteins resulting in neuronal loss [1]. It results in a chronic acquired decline sufficient to affect daily life in at least two of the following domains: memory, reasoning, complex tasks, visuospatial, language, and personality [1,2].

Dementia is the fifth leading cause of death among Americans older than 65 and the seventh among all adults. The lifetime risk of dementia is approximately 17%, with the incidence doubling each decade after 60 years of age. In addition, by 2050, the estimated number of Americans living with dementia will increase from 5 million to 14 million [2].

Alzheimer’s disease (AD) is the most common major neurocognitive disorder accounting for 60-70% of dementia cases worldwide [3]. Surprisingly, dementia with Lewy bodies is the second most common, with 15% of the cases, followed by vascular dementia, with 10% occurrence, and frontotemporal dementia, the less common subtype. Therefore, diagnosis of dementia should focus on comprehensive patient history, neurological examination, and a cognitive assessment. In addition, laboratory tests and neuroimaging studies are often needed to rule out medical conditions that might be contributing to cognitive impairment [3,4].

Unfortunately, the treatment available for AD is limited. Pharmacotherapy does not reverse the disease, but it may slow the progression of dementia. Anti-dementia medication classes include acetylcholinesterase inhibitors (AChEIs), such as donepezil, galantamine, and rivastigmine, N-methyl-D-aspartate (NMDA) receptor antagonist, including memantine, and, most recently, the newly approved monoclonal antibody against amyloid-beta (Aβ), aducanumab [5].

## Review

Medications approved for Alzheimer’s disease

AChEIs are indicated in mild-to-moderate AD. They are also used for mild-to-moderate dementia with Lewy bodies and vascular dementia. This class of medication reversibly inhibits cholinesterase to increase the concentration of acetylcholine at the synaptic gap, which has been shown to improve symptoms modestly in the most common subtypes of dementia. Adverse effects include nausea, vomiting, dizziness, insomnia, cholinergic crisis, anorexia, bradycardia, and conduction abnormalities such as prolongation of the QT interval on the electrocardiogram. Therefore, therapy should not be continued in those without evidence of improvement or significant side effects [6]. The proof of response to AChEIs has been quite variable, thus underscoring the importance of individualized decision-making [7-9]

The NMDA receptor antagonist memantine is indicated in moderate-to-severe AD and is often used concurrently with AChEI. Memantine binds to NMDA receptors, acting as a noncompetitive antagonist that blocks the activity of glutamate, thereby decreasing glutamate-induced calcium-mediated excitotoxicity. Adverse effects include headaches, dizziness, confusion, hallucinations, seizures, and constipation [10].

The Aβ monoclonal antibody aducanumab has been approved by the Food and Drug Administration (FDA) for mild AD using the accelerated approval pathway. It has been claimed to decrease Aβ plaque formation by crossing the blood-brain barrier and selectively targeting and binding aggregated soluble oligomers and insoluble fibrils conformation of Aβ plaques in the brain [11]. Structural analyses have shown that aducanumab binds to a linear epitope formed by Aβ amino acids 3 to 7. In addition, it binds to the N-terminus of Aβ in an extended conformation and is only available through the intravenous route [12].

Conventional versus new therapy

AChEI therapy and NMDA receptor antagonist are generally considered short-term interventions designed to treat the symptoms of AD, such as improvement in executive function, overall cognition, and activities of daily living (ADLs). They do not address AD’s primary pathological mechanisms: 1) extracellular amyloid-beta plaques and 2) intracellular neurofibrillary tangles comprised of abnormal tau protein [13]. In addition, it is not known to be disease-modifying or neuroprotective, with little impact on the actual course or progression of the disease. However, available data suggest that beneficial effects can be maintained for more than 12 months and up to 5 years with these existing agents [14,15].

Aducanumab is the first disease-modifying therapy approved by the FDA and the first AD drug approved since 2003. It is preceded by a multitude of drugs that have attempted to target Aβ, including five anti Aβ antibodies, all of which failed to demonstrate clinical efficacy in their trials [5,16]. Aducanumab’s mechanism of action is unique in its ability to selectively target Aβ aggregates, a core mechanism for AD development. In an early phase of its trial, amyloid plaques were decreased in all examined brain regions after the administration of aducanumab. However, its clinical effects were not shown to be significant [17,18]. Still, these early findings alluded to the potential of aducanumab. They spurred a plethora of research, ultimately culminating in the drug’s approval in June 2021 through an unconventional and controversial process, which will be explored further. The controversy invites scrutiny of aducanumab’s purported benefits and whether it truly outweighs the risks. We have aimed to answer this question based on three factors: 1) cost-effectiveness, 2) potential side effects and monitoring, and 3) efficacy.

Cost-Effectiveness 

The discussion of cost-effectiveness regarding AD is complicated, given that most medications approved for AD treatment are for mild-to-moderate disease, yet 75% or more of healthcare costs associated with AD occur during the severe stages [19]. For example, aducanumab has only been approved for mild cognitive impairment (MCI) and mild AD. Still, studies have stated that delaying the onset and progression of AD by two years can result in a 22.5% reduction in the global disease burden by the year 2050 [20]. However, the cost and specific recommendations for using aducanumab (Table 1) remain a significant barrier to achieving such a goal.

**Table 1:** Recommendations for the use of aducanumab. [21] MCI, mild cognitive impairment; AD, Alzheimer’s dementia; MMSE, Mini-Mental State Examination; MoCA, Montreal Cognitive Assessment; PET, positron emission tomography; CSF, cerebrospinal fluid; MRI, magnetic resonance imaging

Recommendations
A clinical diagnosis of MCI due to AD or mild-stage AD dementia after a comprehensive evaluation
Performance scores of 21 or higher on MMSE or an equivalent validated test such as MoCA with scores of 17 and above
Amyloid positive by PET or an AD signature pattern on CSF testing
Stable medical and cardiovascular/cardiopulmonary conditions
No organ failure or active cancer (low-grade basal and squamous cell carcinomas excepted)
Psychiatrically stable
Not on anticoagulants and no coagulopathy present
Can be on acetylcholinesterase inhibitors and memantine
Baseline MRI with no evidence of acute or subacute hemorrhage, macrohemorrhage, more significant than four microhemorrhages
Baseline MRI with no evidence of cortical infarction (>1.5 cm), one lacunar infarction (>1.5 cm), >1 area of siderosis, or diffuse white matter disease

Biogen, a biotechnology company that conducted the aducanumab controlled trial, initially listed the annual cost at $56,000 per person but received considerable feedback regarding its staggering cost; therefore, the amount was reduced to $28,000 annually at the beginning of 2022 to make it more accessible. However, the total cost is still projected to be higher, given the testing and monitoring needed before and during treatments [22]. Per the Institute for Clinical and Economic Review, a fair annual price would lie somewhere between $2,500 and $8,300. When considering the potential health gains, the most optimistic cost-effectiveness scenario would cost a yearly price of $11,100 to $23,100 [23]. The list price of $28,000 per year still easily exceeds the best-case scenario projection, making it inaccessible for many AD patients.

The cost-effectiveness of aducanumab was further explored in a study that used a Markov model to compare the combination of aducanumab and supportive care versus supportive care alone. It showed that patients treated with aducanumab gained four additional months in earlier stages of AD, resulting in 0.154 more quality-adjusted life-years (QALYs). The incremental cost-effectiveness ratio (ICER) for both the healthcare sector and societal perspective relative to standard care were $1,330,000/QALY and $1,270,000/QALY, respectively. This study was based on aducanumab’s original list price of $56,000 per year [24]. The authors concluded that patients received minimal improvements in health outcomes at a high cost.

Existing research suggests that conventional therapies with AChEIs and memantine are more cost-effective compared to aducanumab and/or the best supportive care. A systematic review of National Health Services data from England and Wales showed a >99% probability that AChEIs are more cost-effective than supportive care alone [25]. A recent study utilizing the Markov model simulated the costs and effectiveness of AChEI-memantine combinations compared with their monotherapies. It showed the rivastigmine transdermal patch resulted in 2.250 QALYs, followed by a rivastigmine-memantine combination with 1.831 QALYs [19].

The rivastigmine patch also demonstrated an ICER of $93,307/QALY, about 10 times less than that of aducanumab. Although the study’s population comprises patients with moderate-to-severe AD, at this time it can be inferred that conventional therapies remain more economical and effective choice for patients and our healthcare system [25].

Side Effects & Monitoring 

An expert panel was structured around detecting amyloid-related imaging abnormalities of the effusion (ARIA-E) and hemorrhagic (ARIA-H) types. Unfortunately, the clinical symptoms suggestive of ARIA and non-ARIA can sometimes be similar, which makes it challenging to differentiate both conditions [21]. Table 2 shows more information about each medication class’s common side effects, incidence, and appropriate monitoring.

**Table 2:** Side effects and monitoring of aducanumab versus AChEIs versus NMDA receptor antagonist GI, gastrointestinal; FLAIR, fluid-attenuated inversion recovery; GRE T2*, gradient-recalled echo-weighted imaging; DWI, diffusion-weighted imaging; AChEIs, acetylcholinesterase inhibitor; NMDA, N-methyl-D-aspartate Clinical applications: T2*-weighted sequences detect deoxygenated hemoglobin, methemoglobin, or hemosiderin in lesions and tissues. You can look at intracranial hemorrhage, arteriovenous malformation, hemorrhage in a tumor, punctate hemorrhages in diffuse axonal injury, and more. Specifically, it is used here to look at the ARIA hemorrhagic type that has been reported with increased frequency in clinical trials of amyloid-lowering therapies such as aducanumab [21].

Side effects and monitoring			
*Drugs*	Aducanumab	Acetylcholinesterase inhibitors	NMDA receptor antagonist
*Adverse effects*	Clinical symptoms suggestive of ARIA-E or ARIA-H include altered mental status, confusion, delirium, disorientation, dizziness, headache, nausea, seizures, vertigo, and visual disturbances. Symptoms typically resolved within 12-20 weeks in clinical trials and have been noted to be dose-dependent. Other risk factors include apolipoprotein E4 carrier status, increasing age, more significant number of microhemorrhages at baseline, cardiovascular risk factors, and/or evidence of a previous cerebrovascular event [26,27].	GI symptoms: upset stomach, nausea, diarrhea, and anorexia. Other side effects: symptomatic bradycardia, rhabdomyolysis, neuroleptic malignant syndrome, and vivid dreams or nightmares [28,29].	Dizziness, confusion, headache, delusion, hallucination, and agitation. Delusions and hallucinations are more common in patients with Lewy body dementia and should thus be avoided in these patients [30,31].
*Incidence*	ARIA-E: brain edema including sulcal effusion (20% to 64%) higher in apolipoprotein E4 carriers. ARIA-H: superficial siderosis (5%) and microhemorrhage (19%). ARIA-E and ARIA-H: falling (15%) and headache (21%). Non-ARIA side effects: diarrhea (9%), altered mental status (≤8%), confusion (≤8%), delirium (≤8%), and disorientation (≤8%) [21,32].	GI symptoms are present in approximately 20-30% of patients taking donepezil. GI symptoms are more prevalent in patients taking galantamine or oral rivastigmine and significantly less prevalent in patients taking transdermal rivastigmine [28,29].	Dizziness (5-7%), confusion (6%), and headaches (6%) are the most common adverse effects. Delusion, hallucinations, and agitation account for only 2-3% of the cases [30,31].
*Monitoring*	MRI obtained at least one year before initiation of treatment or at baseline if there are any suggestions of a focal brain event since the last MRI. MRI was obtained prior to the fifth, seventh, and 12th infusions. Consider additional MRI before the 10th dose (after three doses of 10 mg/kg have been administered) to maximize the detection of ARIA. MRI studies for ARIA should include FLAIR, GRE T2, and quick DWI. If ARIA is symptomatic, suspend treatment and repeat MRIs monthly. If symptoms resolve, treatment can be resumed. Patients with severe symptoms such as seizure or stroke-like syndromes should permanently discontinue aducanumab use [21].	Patients should be monitored periodically at the clinician’s discretion for clinical response and tolerability of medication [33].	Patients should be monitored periodically at the clinician’s discretion for clinical response and tolerability of medication [33].

Efficacy 

Although AChEI therapy and NMDA receptor antagonist are not known to be disease-modifying, their modest efficacy in reducing neuropsychiatric symptoms can still benefit the patient, caregiver, and healthcare system. A meta-analysis of 29 trials showed AChEI therapy to minimize functional impairment for ADLs and instrumental activities of daily living (IADLs) in patients with mild-to-moderate AD [6]. It does not necessarily improve cognition itself but may stabilize or slow the cognitive decline in mild-to-moderate AD [34]. The DOMINO-AD trial also showed that discontinuing AChEI increased the chance of nursing home placement within the first year [35].

For patients with moderate-to-severe AD, memantine as a monotherapy improved scores for cognitive function, behavioral disturbances, ADLs, and global conditions [36]. In combination with an AChEI, memantine showed marginal benefit in cognitive function but was superior in AD with behavioral disturbances. Of the AChEI treatments available, donepezil monotherapy in combination with memantine appear to offer a more significant improvement of the patient’s global condition and with fewer adverse events [34]. Although these interventions are generally regarded as a short-term solution, newer data suggest stabilization of disease can last upwards of 12 or more months [14,15].

The efficacy of aducanumab cannot be discussed without first reviewing its controversial FDA approval. During preclinical trials, aducanumab showed evidence of reducing A𝛃 plaques and reestablishing neuronal calcium permeability, alluding to both disease-modifying and neuroprotective qualities. However, it was not shown to prevent the formation of new Aβ plaques relative to control [17]. Despite this, results were deemed promising, and aducanumab was moved to clinical trials in 2011 [37].

A double-blind, randomized controlled phase Ib clinical trial in 2012 called Prime was the first to show a significant reduction of Aβ plaques in patients with MCI or mild AD and a clinical benefit with a decreased rate of progression of cognitive symptoms [17,37]. However, patients were also shown to have an increased risk of ARIA-E dependent on the administered dose. For example, a staggering 41% of participants receiving 10 mg/kg developed ARIA-E versus 6% of participants receiving 3 mg/kg of aducanumab [17].

Biogen initiated two identical double-blind, randomized controlled phase III clinical trials for aducanumab in 2015 titled ENGAGE and EMERGE with a total of 3,285 enrolled participants from 20 countries [32,37]. The patients were diagnosed with MCI or mild AD and received either low-dose or high-dose aducanumab every four weeks for 18 months. The primary efficacy endpoint of both trials was to demonstrate decreased cognitive decline [17].

However, Biogen terminated both trials early in March 2019 due to concern that the trials were not likely to reach their efficacy endpoint [32]. The biotechnology company collected an additional three months’ worth of data after realizing that the EMERGE trial would have demonstrated favorable results if its data were analyzed independently of the ENGAGE trial [32,37,38]. The supplemental data did indeed show that the high-dose arm of EMERGE met its primary efficacy endpoint, but its clinical significance was questionable. After post-hoc analysis, the ENGAGE trial has still been deemed a failure [38].

In November 2020, Biogen applied for approval of aducanumab with the FDA’s Peripheral and Central Nervous System Drugs Advisory Committee. Ten of 11 members voted against it with one abstention, citing safety concerns with aducanumab’s increased risks of ARIA-E and the discrepant results in its clinical trials [37]. Despite the committee’s decision, FDA approved it in June 2021 via its Accelerated Approval Program. Three drug advisory committee members resigned in protest over the FDA’s decision [39]. At the same time, the FDA’s Acting Commissioner, Janet Woodcock, MD, asked for an independent investigation regarding the interactions between Biogen executives and FDA officials stemming back to 2019 [40].

Furthermore, in December 2021, the European Medicines Agency (EMA) and the Pharmaceuticals and Medical Devices Agency of Japan (PMDA) also denied the approval for aducanumab, citing unsatisfactory results and safety concerns [41]. At the time of press, EMA is reviewing Biogen’s appeal against its decision, while PMDA has requested additional data, which is currently being collected. The Accelerated Approval Program requires ongoing studies known as phase IV confirmatory trials to determine further if the drug provides a clinical benefit or not. Biogen has named its trial ENVISION and is slated for completion in 2026 [37].

## Conclusions

Currently, it is not possible to establish the efficacy of aducanumab, in addition to its questionable approval process by the FDA. As it stands now, aducanumab’s risks outweigh the benefits. The first disease-modifying therapy approved by the FDA is more expensive when compared with conventional treatment and requires rigorous monitoring before, during, and after treatment. In addition, it is only available through the intravenous route and may result in significant clinical symptoms associated with ARIA-E, ARIA-H, and non-ARIA side effects.

Conventional therapies are only for symptom management but are still beneficial to the patient and caregiver. AChEIs and NMDA receptor antagonist are more cost-effective than aducanumab and supportive care.

Given the promise of aducanumab’s potential disease-modifying capabilities, several studies are exploring other pathways that could also be disease-modifying by removing A𝛃 plaques, or reducing the amount of A𝛃 plaques produced in the brain, reducing inflammation, or keeping tau proteins from tangling.
